# Germinal mutations among patients with breast cancer in Colombia: is BRCA3 coming?

**DOI:** 10.3332/ecancer.2025.1859

**Published:** 2025-02-27

**Authors:** Lisa Ximena Rodríguez Rojas, Liliana Doza Martínez, Jorge Andrés Olave Rodríguez, Sandra Eliana Murillo Rusynke, Paola Andrea Pérez Castellano, David Alexander Bolaños Beltrán, Helen Johana Ortiz Rojas, José Antonio Nastasi Catanese

**Affiliations:** 1 Fundación Valle del Lili, Servicio de Genética Humana, Cra 98 No. 18 - 49, Cali 760031, Colombia; 2 Facultad de Ciencias de la Salud, Universidad ICESI, Cali 760031, Colombia; 3 Fundación Valle del Lili, Centro de Investigaciones Clínicas, Cra 98 No. 18 - 49, Cali 760031, Colombia

**Keywords:** BRCA1, BRCA2, BRCA3, ATM, germ mutation, multigene panel, hereditary breast cancer

## Abstract

**Purpose:**

Breast cancer is the most common type of cancer in women and accounts for 25% of all cancers worldwide. The mechanisms by which it develops include germline (generally inherited) and somatic mutations. There are six mutations with the highest incidence in the Colombian population, called the Colombia profile, which is associated with the *BRCA1* and *BRCA2* genes. The aim of this study is to identify germline mutations in individuals with breast cancer, such as BRCA and other genes.

**Methods:**

This study describes the frequency and type of variants in hereditary cancer genes associated with breast cancer detected by the next-generation sequencing of a panel of 111 hereditary cancer genes, including *BRCA1* and *BRCA2*.

**Results:**

This analysis allowed the identification of variants associated with breast cancer in 307 patients from a population in southwestern Colombia, of which 19% had pathogenic and probably pathogenic mutations associated with hereditary cancer. According to the variant classification, it was found that the mutation frequency in BRCA1 was 17%, in BRCA2 was 14% and in the *ATM* gene was 12%; nevertheless, 57% of mutations were attributed to other genes such as *MUTYH, FANCM, FANCA* and* TP53*. Four patients were found to have the mutation c.3450delCAAG in the *BRCA1* gene, which is included in the Colombia profile.

**Conclusion:**

In summary, in the Colombian population, there is a great diversity of germline mutations in genes other than *BRCA1* and *BRCA2 *that are associated with breast cancer. Studying mutations and variants of uncertain significance in *ATM* could improve understanding of how mutations in these genes contribute to cancer and whether *ATM* should be considered as *BRCA3*.

## Introduction

Breast cancer is one of the leading causes of death worldwide in the female population, with an estimated 2,600,000 cases and 685,000 deaths in 2022 [1]. In Colombia, the age-standardised incidence rate remained relatively stable between 2012 and 2020 (43.1–47.8 cases per 100,000 women-years). Additionally, survival since 1995 has presented a substantial improvement from 65.7 to 72.1 [2].

The gene alterations can be inherited or acquired after exposure to carcinogenic agents. Risk factors such as hormonal regulation and lifestyle have also been identified [3–5]; however, heredity represents one of the most important risk factors [6, 7]. It is estimated that 5%–10% of breast cancers are attributable to hereditary syndromes with autosomal dominant transmission, such as hereditary breast and ovarian cancer syndrome (HBOC). The *BCRA1* and *BCRA2* genes are responsible for 30%–50% of HBOC cases [6, 7].

The individual risk of breast cancer increases proportionally with the number of affected relatives, early age of onset, biopsy with atypical hyperplasia, biopsy with lobular or ductal carcinoma *in situ*, reproductive history, early menarche, late menopause and use of oral contraceptives [3, 4]. Concerning somatic factors, the activation of dominant oncogenes involved in the proliferation of tumor cells, along with the inactivation of recessive tumor suppressor genes, is often due to genetic and epigenetic alterations where the loss of function fosters malignancy [3–5]. Many of the mutations in these genes cause inherited cancer; among those associated with breast cancer are *BRCA1, BCRA2, ATM, TP53, PTEN, STK11, CDH1, MLH1, MSH2, MSH6, PMS1, PMS2, FANCE, FANCF* and *FANCG* [2]. HBOC is a condition that increases the probability of developing breast, ovarian and other types of cancer, such as pancreatic and prostate cancer, due to the presence of germline mutations in genes of greater susceptibility, such as *BRCA1* or *BRCA2* [9–12].

In 2007, two recurrent BRCA1 mutations were reported in Colombia: 3450 delCAAG and A1708E, which accounted for 100% of all BRCA1 mutations identified in this cohort. Meanwhile, the recurrent BRCA2 mutation 3034 delACAA represented 40% of all BRCA2 mutations. Haplotype analyses indicated that each of these mutations originated from a common ancestor. This group of mutations has been termed the Colombia profile; with the authors suggesting that the high percentage of recurrent mutations: 85% of all mutations identified in this cohort may facilitate carrier detection in the Hispanic population of Colombia [13]. Numerous systematic reviews and meta-analyses have identified a link between ATM variants and an increased risk of breast cancer, establishing this gene as the third most common cause of hereditary cancer across different populations [14–17]. In this country, it is important to carry out studies that allow the characterization of the population. The aim of this study was to describe and identify germline variants linked to breast cancer in patients with breast cancer, particularly those with early-onset presentation and/or a family history of cancer, within a highly complex institution in southwestern Colombia (Fundación Valle del Lili, Cali-Colombia). Several pathogenic variants were identified, and only one of those noted in the Colombia profile indicated that the population displays significant genetic heterogeneity. The identification of the profile of the population allows an approach to the knowledge gap of the disease, which provides a focus on diagnosis, the development of timely therapies and a treatment towards personalised medicine.

## Materials and methods

**Figure 1.**
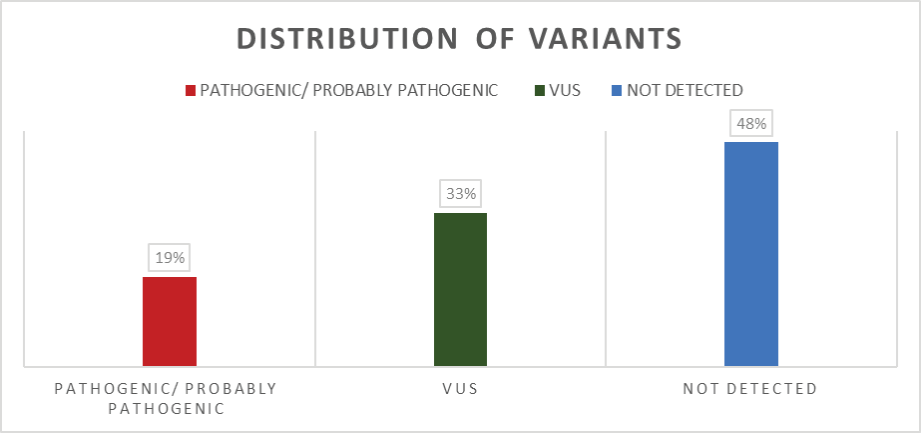
Distribution of variants according to their classification in the population studied.

A retrospective cross-sectional study was conducted, in which 307 patients with a previous diagnosis of breast cancer derived from the Oncogenetic unit of the Fundación Valle del Lili in Cali, Colombia, were analysed; 96.4% of patients were diagnosed with invasive ductal carcinoma (*n* = 291), while 2.6% (*n* = 8) were diagnosed with invasive lobular carcinoma. All patients received pre- and post-test genetic counseling in our institution. All patients included in the study were tested for a commercial genetic panel of hereditary cancer of 111 genes by next-generation sequencing (NGS). The study was approved by the hospital ethics committee. A bioinformatics analysis was performed with in silico tools and a bibliographic search for the classification of the variants according to the criteria of the American College of Medical Genetics and Genomics (ACMG), and these were classified as pathogenic, probably pathogenic, variants of uncertain significance (VUS), probably benign and benign (Figure 1). These last two categories of variants were not included in this report. The pathogenic or probably pathogenic variants were confirmed by Sanger sequencing.

### Data analysis

This research was carried out for convenience within the framework of a descriptive and analytical study that allowed us to analyse the germline genomic variants associated with breast cancer found in a population of southwestern Colombia. These were recorded in Excel^®^ tables where a tabulation of the variants according to their genomic position, nucleotide change, amino acid change, clinical significance, allelic frequency and family history was performed, with the help of databases of DNA sequences provided by the National Center for Biotechnology Information, taking into account the classification of the ACMG, which allowed the results to be analysed. Statistical analysis of the data was performed using Stata v14.0.

## Results

In total, 307 patients with breast cancer and suspected hereditary cancer syndrome HBOC were treated at the Oncogenetics Unit of Fundación Valle del Lili, a reference center in the southwest of Colombia over a 2-year period. The mean age of the participants in the study was 45 years (SD = 10.2). It was found that 78% of patients carrying P, PP and VUS variants had a family history of cancer in at least one close first-degree relative (Table 1). All of them underwent a multigene NGS panel for hereditary cancer, and 160 variants were detected in 160 patients. It was found that 59 patients (19%) (Figure 1, Table 2) carried a deleterious germline variant, classified as pathogenic or probably pathogenic, in genes associated with hereditary cancer that could be involved in the development of breast cancer: 10 in *BRCA1*, 8 in *BRCA2*, 7 in *ATM*, 3 in *CHEK2,* 3 in *TP53*, 4 in *MUTYH*, 3 in *FANCA* and 4 in *FANCM*, among others. In 101 patients (33%), variants of uncertain significance were detected in different genes associated with cancer, such as *BRCA1, BRCA2, APC, ATM, FANCA, FANCM, POLE, RAD50* and *RAD51,* among others, and no variant was detected in the 147 remaining patients (48%) (Figure 1). We found two mutations not previously reported in the literature in genes considered to be of moderate penetrance: *RAD50* (NM_005732.4): c.1728del and *WRN* (NM_000553.6): c.464T> A.

**Figure 2.**
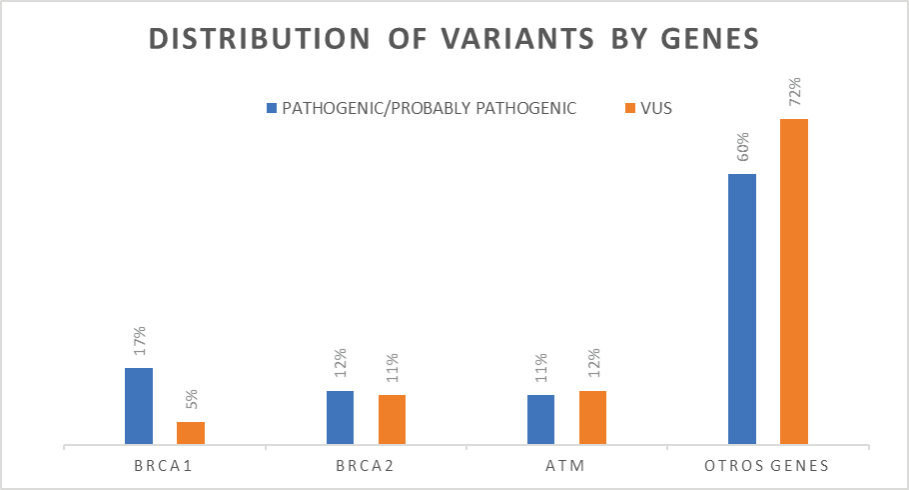
Distribution of variants by genes.

In the BRCA1 and BRCA2 genes, pathogenic and probably pathogenic variants were identified in 17%–14%, respectively; in the ATM gene, 12% of pathogenic and likely pathogenic mutations were detected. Deleterious variants were also found in other genes, accounting for 57% of all mutations identified (Figure 2).

Patients with breast cancer with deleterious variants associated with the *BRCA1* and *BRCA2* genes had a mean age of 43 years (SD = 11.71) and 41 years (SD = 14.62), respectively. Of the 18 patients with mutations in the BRCA1 and BRCA2 genes, 14 had a family history (FH) of cancer; of the 7 patients with mutations in the ATM gene, 5 had a cancer FH, and of the 34 patients with other altered genes, 27 had a cancer FH. Furthermore, among the 101 patients with identified variants of uncertain significance, 79 had a family history of cancer (Table 1). Of the 18 patients with BRCA2 variants, 15 did not express Her2 (Her2-), while of the 15 cases with BRCA1 variants, 12 also did not express Her2 (Her2-). Patients with BRCA1 variants showed the highest number of cases lacking expression of ER (ER-) and EP (EP-). The ATM group had the highest number of HER2-positive cases (Table 1).

In our study, one of the variants included in the ‘Colombia Profile’, c.3450delCAAG, was found in four patients. A high frequency of other variants not described in this Profile was found; for example, the c.1674delA variant of *BRCA1* was detected in 4 out of 10 patients, and the c.6275_6276delTT variant of the *BRCA2* gene was detected in 5 out of 8 patients.

**Figure 3.**
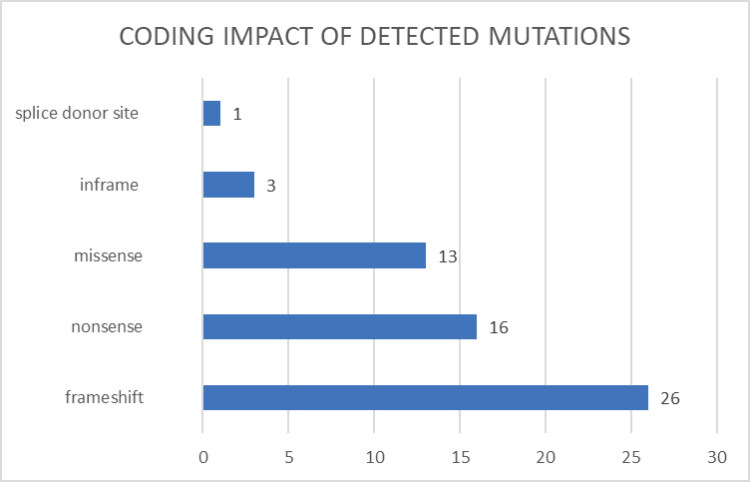
Coding impact of detected mutations.

Among the mutations detected, we found that 44% were frameshift mutations (26 cases), 27% were nonsense mutations (16 cases), 22% were missense mutations (13 cases), 5% were inframe mutations (3 cases) and 2% affected the splicing site (1 case) (Figure 3).

Among the VUS, 95% of the variants detected were missense (96 cases), 3% were synonymous (3 cases) and 2% were inframe mutations (2 cases).

In total, 46 of 59 patients with pathogenic and/or probably pathogenic mutations had a family history of cancer, and 79 of 101 patients with VUS also had a family history of cancer.

## Discussion

We assessed the occurrence of germline mutations in genes linked to hereditary cancer in patients with breast cancer treated at the Oncogenetics Unit of Fundación Valle del Lili. Nineteen percent of our population with breast cancer presented mutations in the genes studied, and 33% of this population carried VUS in these genes. The frequency of mutations in the *BRCA1* and *BRCA2* genes was 31%. We noted that the ATM gene exhibited an 11.6% frequency of deleterious mutations and a mutation frequency of 57% was observed in the other genes studied. These data are very similar to those of other studies carried out in the Hispanic population [8, 13, 18]. Other genes that showed deleterious variants were *MUTYH* (6.6%), *FANCM* (6.6%), *FANCA* (5%), *TP53* (5%) and other genes (35%). Similar to other studies, the third most frequently mutated gene in our study was *ATM*. The *ATM* gene shares functional similarities to the *BRCA1* and *BRCA2* genes: it is involved in the DNA damage response and plays an important role in double-stranded DNA repair. It has also been observed that heterozygous mutations in the *ATM* gene are associated with an increased risk of developing breast cancer. With these associations, the designation of *ATM* as *BRCA3* (Breast Cancer 3) could be suggested; in fact, greater information and clinical management guidelines are already included in the National Comprehensive Cancer Network^®^ (NCCN) guidelines for mutations in this gene. Of the mutations described as founding in Colombia by Briceño-Balcázar *et al*. [8], we found a low frequency (4/18, 22%) compared to what was previously reported in Colombia (up to 77%) [13]. Additionally, only one of them was observed (c.3450delCAAG). It is possible that mutations previously reported in Colombia are common in some specific subpopulations, and this genetic variability may be due to factors such as historical migration, population mix and geographic isolation. Likewise, we observed other mutations in the *BRCA* genes with equal or greater frequency, such as c.1674del (4/18) in *BRCA1* and c.6275_6276del (5/18) in *BRCA2*. As sequencing technology and methods advance, new mutations are discovered in different genes, including *BRCA1* and *BRCA2.* NGS has allowed us to analyse genes in greater detail and find mutations that were previously unknown, went unnoticed or were not included in the reports. The great information provided by NGS has made it possible to expand the databases, facilitating the identification and characterization of new mutations. We found a frequency of 16% of VUS in the *BRCA* genes, similar to that reported in the current literature between 10% and 20% [19], 12% of VUS in ATM and 72% of VUS in other genes. VUS are genetic changes with unknown pathogenic impact. Our study demonstrates the need to expand the number of genes studied in patients with breast cancer who meet the NCCN criteria for hereditary cancer, although, as is already known, the greater number of genes studied increases the number of VUS detected. These variants represent a challenge in the interpretation of the results of genetic studies and can generate confusion and anxiety in the people who carry them. Properly reviewing and classifying these variants is essential to providing accurate and up-to-date genetic counseling to patients and their families. This involves determining whether additional screening measures are needed, such as more frequent medical examinations or preventive interventions. In our center (Fundación Valle del Lili), we follow up 1-2 times a year to review the variants detected in the panel, the literature and the databases and thus know if they have been reclassified or if there is a greater tendency towards pathogenicity. Likewise, new cases are reviewed in the families of VUS carriers to carry out segregation studies and try to give clinical significance to this variant in the family. Multigene panels such as the one analysed in this study include high, medium and low penetrance genes, which could lead to incidental findings not related to the expected syndrome, but we also consider that it contributes to the knowledge and, perhaps, to the discovery of new genes and/or new causal gene–disease relationships. This study reveals distinct patterns in the molecular profiles of breast tumors linked to mutations in BRCA1, BRCA2 and ATM. A high prevalence of Her2-negative tumors was noted in patients with BRCA1 and BRCA2 variants, with BRCA1 also showing a strong association with the triple-negative phenotype as demonstrated in prior studies [20]. In contrast, mutations in ATM were linked to a high prevalence of Her2-positive tumors, an interesting finding considering that Her2+ tumors typically account for only 20%–30% of all breast cancers. Bassi *et al* [21] highlighted the significance of the PTEN-ATM axis in regulating the G1/S cell cycle checkpoint, where PTEN must be phosphorylated by ATM. They demonstrate that a mutated PTEN cannot undergo phosphorylation by ATM, which accelerates tumorigenesis in Her2+ breast tumors. Based on these findings, it can be suggested that a mutated ATM, which fails to phosphorylate PTEN, would promote tumorigenesis in this type of cancer (Her2+ breast cancer). This may explain the prevalence of Her2+ breast tumors in patients with mutated ATM [21].

These findings emphasise the importance of comprehensive molecular characterization of breast tumors, including not only the status of BRCA1/2 but also other susceptibility genes such as ATM. Identifying these mutations and their associations with specific molecular profiles could significantly improve risk stratification and the selection of personalized treatments. Further research is required to fully understand these relationships and their prognostic and therapeutic implications, particularly regarding the association between ATM mutations and Her2 expression.

We found two mutations not previously reported to our knowledge: *RAD50* (NM_005732.4): c.1728del and *WRN* (NM_000553.6): c.464T>A. These two genes, considered of moderate penetrance, such as *RAD50*, can contribute to inherited cancer by altering DNA repair mechanisms, increasing the risk of accumulation of genetic mutations and, ultimately, the risk of cancer development in certain individuals. Among the most frequent mutations detected, we found that 71% had a frameshift and nonsense impact (42 cases). Although these two types of mutations may be frequent in hereditary cancer studies, this may depend on the specific gene being analysed and the population studied. Additionally, other mutations, such as missense variants, may also be relevant in the context of hereditary cancer. In this study, 22% of the mutations were missense (13 cases), and 95% of the VUS were also classified as missense. The interpretation of genetic variants is a complex and challenging process; missense variants can have an uncertain or ambiguous impact on protein function. Information on the functional consequences of these variants can be limited or contradictory on many occasions. This may lead to a greater number of missense variants in the reports of multigene studies because their significance may require a more detailed evaluation and further functional or clinical analysis. The adequate interpretation of these variants requires a comprehensive approach that considers the clinical, functional and population evidence, as well as the clinical context of the patient and within the framework of genetic counseling carried out by an expert in oncogenetics.

## Conclusion

The present study of 307 patients with breast cancer revealed germline mutations in 19% of cases. BRCA1/2 genes accounted for 31% of pathogenic mutations, while the ATM gene represented 12%. The high frequency of ATM mutations (12% overall) and its functional similarity to BRCA1/2 suggest it could be considered as BRCA3. Of the 307 patients with breast cancer, 33% had VUS, primarily in ATM (12%), BRCA2 (11%) and BRCA1 (5%). The majority of VUS (95%) were missense mutations. Investigating VUS, especially in ATM, is essential for understanding cancer predisposition mechanisms, developing targeted therapies and potentially recognizing ATM as BRCA3 due to its functional similarities with BRCA1/2. Carriers of uncertain variants require thorough follow-up, including segregation and functional studies, to properly classify these variants. Expert oncogenetic counseling is crucial. Multigene panel testing facilitates the discovery of new variants, potentially revealing novel gene-disease associations.

## Conflicts of interests

The authors declare that they have no conflicts of interest during the conduct of this study.

## Funding

The authors declare that they did not receive funds or other support during the preparation of this manuscript.

## Consent to participate

Informed consent was obtained from all individual participants included in the study.

## Author contributions

All authors contributed to the conception, study design, material preparation, data collection and analysis. All authors read and approved the final manuscript.

## Data availability

Data supporting the findings of this study are available from the corresponding authors.

## Ethical approval

All procedures performed with human participants were in accordance with the ethical standards of the institution and with the Declaration of Helsinki of 1964 and its subsequent modifications or comparable ethical standards.

## Figures and Tables

**Table 1.** Family history of cancer, receptors and detected variants.

		BRCA1	BRCA2	ATM	Other genes	Total
Pathogenic/ probably pathogenic variants *n* = 59	Number of patients with P/PP variant	10	8	7	34	59
	family history of cancer	8 (80%)	6 (75%)	5 (71%)	27 (77%)	46 (78%)
Variants of uncertain significance *n* = 101	Number of patients with VUS	5	11	10	74	101
	family history of cancer	0 (0%)	9 (82%)	8 (80%)	62 (83%)	79 (78%)
Estrogen receptor positive		4	11	10	68	
Estrogen receptor negative		10	6	6	33	
Progesterone receptor positive		4	10	10	68	
Progesterone receptor negative		10	7	6	33	
HER2 positive		2	2	6	26	
HER2 negative		12	15	9	72	

**Table 2.** Mutations detected in the population studied.

Gen	Genetic location	Change in DNA	Protein change	Zygosity	Variant classification	Variant type	Family history
*AIP*	11q13.2	(NM_003977.4):c.646G>T	p.(Glu216Ter)	Heterozygous	Likely Pathogenic	Nonsense	Yes
*ATM*	11q22.3	(NM_000051.4):c.43del	p.(Leu15Terfs)	Heterozygous	Likely Pathogenic	Frameshift	No
*ATM*	11q22.3	(NM_000051.4):c.5690del	p.(Phe1897SerfsTer20)	Heterozygous	Likely Pathogenic	Frameshift	No
*ATM*	11q22.3	(NM_000051.4):c.3673C>T	p.(Gln1225Ter)	Heterozygous	Pathogenic	Nonsense	Yes
*ATM*	11q22.3	(NM_000051.4):c.7767del	p.(Lys2589AsnfsTer17)	Heterozygous	Pathogenic	Frameshift	Yes
*ATM*	11q22.3	(NM_000051.4):c.5074A>T	p.(Lys1692Ter)	Heterozygous	Likely Pathogenic	Nonsense	No
*ATM*	11q22.3	(NM_000051.4):c.2023C> T	p.(Gln675Ter)	Heterozygous	Pathogenic	Nonsense	Yes
*ATM*	11q22.3	(NM_001351836.1):c.43del	p.(Leu15Ter)	Heterozygous	Pathogenic	Nonsense	Yes
*BRCA1*	17q21.3	(NM_007294.3):c.1674del	p.(Gly559ValfsTer13)	Heterozygous	Pathogenic	Missense	Yes
*BRCA1*	17q21.31	(NM_007300.3):c.3331_3334del	p.(Gln1111AsnfsTer5)	Heterozygous	Pathogenic	Frameshift	Yes
*BRCA1*	17q21.31	(NM_007300.3):c.3331_3334del	p.(Gln1111AsnfsTer5)	Heterozygous	Pathogenic	Frameshift	Yes
*BRCA1*	17q21.31	(NM_007300.3):c.3331_3334del	p.(Gln1111AsnfsTer5)	Heterozygous	Pathogenic	Frameshift	Yes
*BRCA1*	17q21.31	(NM_007300.4):c.1674del	p.(Gly559ValfsTer13)	Heterozygous	Pathogenic	Frameshift	No
*BRCA1*	17q21.31	(NM_007300.3):c.3331_3334del	p.(Gln1111AsnfsTer5)	Heterozygous	Pathogenic	Frameshift	No
*BRCA1*	17q21.31	(NM_007300.4):c.5093_5096del	p.(Thr1698IlefsTer2)	Heterozygous	Pathogenic	Frameshift	Yes
*BRCA1*	17q21.31	(NM_007300.4):c.1674del	p.(Gly559ValfsTer13)	Heterozygous	Pathogenic	Frameshift	Yes
*BRCA1*	17q21.31	(NM_007300.4):c.1674 del	p.Gly559ValfsTer13	Heterozygous	Pathogenic	Frameshift	Yes
*BRCA1*	17q21.31	(NM_007294.4):c.5177_5180del	p.(Arg1726LysfsTer3)	Heterozygous	Pathogenic	Frameshift	Yes
*BRCA2*	13q13.1	(NM_000059.4):c.9246dup	p.(Lys3083GlufsTer28)	Heterozygous	Pathogenic	Frameshift	Yes
*BRCA2*	13q13.1	(NM_000059.3):c.6275_6276del	p.(Leu2092ProfsTer7)	Heterozygous	Pathogenic	Frameshift	Yes
*BRCA2*	13q13.1	(NM_000059.3):c.7673_7674del	p.(Glu2558ValfsTer7)	Heterozygous	Pathogenic	Frameshift	No
*BRCA2*	13q13.1	(NM_000059.3):c.6275_6276del	p.(Leu2092ProfsTer7)	Heterozygous	Pathogenic	Frameshift	No
*BRCA2*	13q13.1	(NM00059.4):c.6275_6276del￼	p.(Leu2092ProfsTer79)	Heterozygous	Likely pathogenic	Frameshift	Yes
*BRCA2*	13q13,1	(NM_000059.4):c.1796_1800del	p.(Ser599Ter)	Heterozygous	Pathogenic	Nonsense	Yes
*BRCA2*	13q13.1	(NM_000059.4):c.6275_6276del	p.(Leu2092ProfsTer79)	Heterozygous	Pathogenic	Frameshift	Yes
*BRCA2*	13q13.1	(NM_000059.4):c.6275_6276del	p.(Leu2092ProfsTer7)	Heterozygous	Pathogenic	Frameshift	Yes
BRIP1	17q23,2	(NM_032043.3):c.1565C>A	p.(Ser522Ter)	Heterozygous	Likely pathogenic	Nonsense	Yes
*CHEK2*	22q12.1	(NM_001005735.2):c.1317del	p.(Val440PhefsTer17)	Heterozygous	Pathogenic	Frameshift	Yes
*CHEK2*	22q12.1	(NM_007194.4):c.520C>T	p.(Leu174phe)	Heterozygous	Likely pathogenic	Missense	Yes
*CHEK2*	22q12.1	(NM_007194.4): c.1095+1G>A	Q.?	Heterozygous	Pathogenic	Frameshift	No
*EPCAM*	2p21	(NM_002354.2):c.394G>T	p.(Glu132Ter)	Heterozygous	Likely pathogenic	Nonsense	Yes
*EPCAM*	2p21	(NM_002354.2):c.745_749del	p.(Ile249LeufsTer3)	Heterozygous	Likely pathogenic	Frameshift	Yes
*ERCC1*	19q13.32	(NM_001983.4):c.180C>G	p.(Tyr60Ter)	Heterozygous	Likely pathogenic	Nonsense	Yes
*ERCC1*	19q13.32	(NM_001983.4):c.467G>A	p8.Arg156Gln)	Heterozygous	Likely pathogenic	Missense	Yes
*ERCC3*	2q14.3	(NM_000122.1):c.325C>T	p.(Arg109Ter)	Heterozygous	Pathogenic	Nonsense	No
*ERCC6*	10q11.23	(NM_000124.4):c.2924G>A	p.Arg975Gln	Heterozygous	Likely pathogenic	Missense	Yes
*FANCA*	16q24.3	(NM_000135.4.3):c.1303C>T	p.(Arg435Cys)	Heterozygous	Pathogenic	Missense	No
*FANCA*	16q24.3	(NM_000135.4):c.3795del	p.(Phe1265LeufsTer4)	Heterozygous	Likely pathogenic	Nonsense	Yes
*FANCA*	16q24.3	(NM_000135.4): c.1115_1118del	p.(Val372alafsTer42)	Heterozygous	Pathogenic	Frameshift	Yes
*FANCM*	14q21.2	(NM_020937.4):c.5893_5895del	p.(Val1965del)	Heterozygous	Likely pathogenic	InFrame	Yes
*FANCM*	14q21.2	(NM_020937.4):c.5893_5895del	p.(Val1965del)	Heterozygous	Likely pathogenic	InFrame	Yes
*FANCM*	14q21.2	(NM_020937.4): c.2586_2589del	p.(Lys863IlefsTer12)	Heterozygous	Likely pathogenic	Frameshift	Yes
*FANCM*	14q21.2	(NM_020937.4):c.2255C>G	p.(Ser752Ter)	Heterozygous	Pathogenic	Nonsense	No
*MLH1*	3p22.2	(NM_000249.3):c.1039-1G>A	p.?	Heterozygous	Pathogenic	Missense	No
*MLH1*	3p22.2	(NM_001354629.1):c.1853_1855del	p.(Lys618del)	Heterozygous	Pathogenic	InFrame	No
*MUTYH*	1p34.1	(NM_001128425.1):c.1187G>A	p.(Gly396Asp)	Heterozygous	Pathogenic	Missense	Yes
*MUTYH*	1p34.1	(NM_012222.2):c.527A>G	p.(Tyr176Cys)	Heterozygous	Pathogenic	Missense	Yes
*MUTYH*	1p34.1	(NM_001128425.1):c.1178G>A	p.(Gly396Asp)	Heterozygous	Pathogenic	Missense	Yes
*MUTYH*	1p34.1	(NM_012222.2):c.1178G>A	p.(Gly393Asp)	Heterozygous	Pathogenic	Missense	Yes
*PMS2*	7p22.1	(NM_001322014.2):c.2243_2246del	(p.Lys748MetfsTer21)	Heterozygous	Likely pathogenic	Frameshift	Yes
*RAD50*	5q31.1	(NM_005732.4):c.1728del	p.(Glu577LysfsTer21)	Heterozygous	Likely pathogenic	Frameshift	Yes
*RAD51*	15q15.1	(NM_002875.5):c.773A>C	p.(Glu258ala)	Heterozygous	Likely pathogenic	Missense	Yes
*RAD51C*	17q22	(NM_058216.3):c.414G>C	p.(Leu138Phe)	Heterozygous	Likely pathogenic	Missense	Yes
*RB1*	13q14.2	(NM_000321.3):c.539+1G>A	p.?	Heterozygous	Likely pathogenic	Splice donor site c.539+1G>A	Yes
*SDHA*	5p15.33	(NM_004168.4):c.964C>T	p.(Gln322Ter)	Heterozygous	Pathogenic	Nonsense	Yes
*TP53*	17p13.1	(NM_001276761.2):c.520C>T	P.(Arg174Ter)	Heterozygous	Pathogenic	Nonsense	Yes
*TP53*	17p13.1	(NM_000546.6):c.586C>T	p.(Arg196Ter)	Heterozygous	Pathogenic	Nonsense	Yes
*TP53*	17p13.1	(NM_000546.6):c.646G>A	p.(Val216 Met)	Heterozygous	Likely pathogenic	Missense	Yes
*WRN*	8p12	(NM_000553.6):c.464T>A	p.(Leu155Ter)	Heterozygous	Likely pathogenic	Nonsense	Yes
